# National Association of Dental Examiners

**Published:** 1892-09

**Authors:** 


### NATIONAL ASSOCIATION OF DENTAL EXAMINERS.

The eleventh annual meeting of the National Association of Dental Examiners was held at Niagara Falls, commencing Monday, August 1, 1892.

The sessions were presided over by the vice-president, Dr. Magill, the elected president, Dr. L. D. Shepard, of Boston, explaining his resignation from the State Board of Massachusetts, which necessarily carried with it his resignation of the presidency of the association. The resignation was accepted with regret, and Dr. Shepard was unanimously accorded the privileges of the floor.

The following State boards were represented at the sessions :

Colorado.—George J. Hartung.

Georgia.—D. D. Atkinson.
Iowa.—J. T. Abbott, J. B. Monfort.
Indiana.—S. T. Kirk.
Maryland.—T. S. Waters.
Minnesota.—L. W. Lyon.
Massachusetts.—E. V. McLeod.
New Jersey.—Fred. A. Levy.
Ohio.—Grant Mollyneaux, Grant Mitchell.
Pennsylvania.—W. E. Magill, Louis Jack, J. A. Libbey.
Tennessee.—J. Y. Crawford.
Wisconsin.—Edgar Palmer.
Kansas.—A. H. Thompson.

The following boards were admitted to membership:

Virginia.—J. Hall Moore.
North Carolina.—V. E. Turner.
Oklahoma.—D. A. Peoples.
South Dakota.—C. W. Sturtevant.
District of Columbia.—Williams Donnally.

At the instance of the committee on colleges, the following communication was sent to the National Association of Dental Faculties:

NIAGARA FALLS, Aug. 1, 1892.

*To the National Association of Dental Faculties:*

GENTLEMEN,—Whereas, a very considerable abuse has arisen by the improper use by students of the various certificates of the schools, such as the "standing" and "passing' certificates, to support students and graduates under age in their attempt to illegally engage in practice; we therefore ask your association to request the various colleges to have their "standing" and "passing" certificates of such uniformity of terms in each case that they can be used for no other purpose, and that they be printed in few words and small type, and be signed only by the dean,

Respectfully,

NATIONAL ASSOCIATION OF DENTAL EXAMINERS,

FRED. A. LEVY, *Secretary.*

A committee of conference was appointed, consisting of Drs Truman, Marshall and Swain, on the part of the Faculties Association, and Donnally, Palmer and Monfort, on the part of the Examiners Association, which after consultation agreed upon a favorable report.

Dr. Lyon offered the resignation of the Minnesota board, which was laid upon the table, as it had evidently been offered as the result of a misunderstanding, and the board was requested to withdraw it.

The following resolution, offered by Dr. Crawford, was adopted.

*Resolved*, That when a member of any State board becomes a teacher of a dental school, his resignation from his board should follow.

A resolution protesting against the classification of dentists as manufacturers and the collection of census statistics from them under the provisions of House Bill No. 7696, commonly known as the Wilcox bill, was adopted. The resolution was similar in terms to those adopted by other dental societies.

The committee on colleges reported that they had received reports showing that the actual number of students in attendance at the last sessions in the schools recognized by the Examiners Association was 2881; of graduates, 1357. In the schools not recognized by the association the students were 236; graduates, 96.

The report also considered desirable advances to be made in educational methods, and offered the following memorial, which the secretary was directed to transmit to the National Association of Dental Faculties :

The National Association of Dental Examiners would respectfully memorialize the National Association of Dental Faculties to authorize two advances in the system of dental education.

These are : First, that your association require the universal enforcement of a higher grade of preliminary education of candidates for matriculation. This proposition lies at the foundation of dental education, in which is involved the quality of the graduates of the future, upon which depend the advancement, the standing, and the dignity of the dental profession.

The second proposition is that complete preparation be made in each school for laboratory technique in the studies of histology, pathology, and in each of the departments of dental surgery and dental prosthesis, and that this method of teaching be made a requirement of the schools.

The committee also reported the following amended list of colleges which they recommend as reputable:

Baltimore College of Dental Surgery, Baltimore, Md.

Boston Dental College, Boston, Mass.

Chicago College of Dental Surgery, Chicago, Ill.

College of Dentistry, Department of Medicine, University of Minnesota, Minneapolis, Minn.

Dental Department, Columbian University, Washington, D. C.

Dental Department, National University, Washington, D. C.

Northwestern University Dental School, (Formerly Dental Department of Northwestern University, University Dental College.)

Dental Department of Southern Medical College, Atlanta, Ga.

Dental Department of University of Tennessee, Nashville, Tenn

Harvard University, Dental Department, Cambridge, Mass.

Indiana Dental College, Indianapolis, Ind.

Kansas City Dental College, Kansas City, Mo.

Louisville College of Dentistry, Louisville, Ky.

Missouri Dental College, St. Louis, Mo.

New York College of Dentistry, New York City.

Northwestern College of Dental Surgery, Chicago, Ill.

Ohio College of Dental Surgery, Cincinnati, O.

Pennsylvania College of Dental Surgery, Philadelphia, Pa.

Philadelphia Dental College, Philadelphia, Pa.

School of Dentistry of Meharry Medical Department of Central Tennessee College, Nashville, Tenn.

University of California, Dental Department, San Francisco, Cal.

University of Iowa, Dental Department, Iowa City, Ia.

University of Maryland, Dental Department, Baltimore, Md.

University of Michigan, Dental Department, Ann Arbor, Mich.

University of Pennsylvania, Dental Department, Philadelphia, Pa.

Vanderbilt University, Dental Department, Nashville, Tenn.

Western Dental College, Kansas City, Mo.

Minnesota Hospital College, Dental Department, Minneapolis, Minn. (defunct).

St. Paul Medical College, Dental Department, St. Paul, Minn. (defunct.)

American College of Dental Surgery, Chicago, Ill.

The report was adopted.

The following officers were elected for the ensuing year: W. E. Magill, Erie, Pa., president; J. Y. Crawford, Nashville, Tenn., vice-president; Fred. A. Levy, Orange, N. J., secretary and treasurer.

Adjourned.

